# Test Characteristics of Urinary Lipoarabinomannan and Predictors of Mortality among Hospitalized HIV-Infected Tuberculosis Suspects in Tanzania

**DOI:** 10.1371/journal.pone.0032876

**Published:** 2012-03-08

**Authors:** Elizabeth Talbot, Patricia Munseri, Pedro Teixeira, Mecky Matee, Muhammad Bakari, Timothy Lahey, Fordham von Reyn

**Affiliations:** 1 Dartmouth Medical School, Hanover, New Hampshire, United States of America; 2 Foundation for Innovative New Diagnostics, Geneva, Switzerland; 3 Muhimbili University of Health and Allied Sciences, Dar es Salaam, Tanzania; 4 Karolinska Institutet, Stockholm, Sweden; National Institute of Allergy and Infectious Diseases, United States of America

## Abstract

**Background:**

Tuberculosis is the most common cause of death among patients with HIV infection living in tuberculosis endemic countries, but many cases are not diagnosed pre-mortem. We assessed the test characteristics of urinary lipoarabinomannan (LAM) and predictors of mortality among HIV-associated tuberculosis suspects in Tanzania.

**Methods:**

We prospectively enrolled hospitalized HIV-infected patients in Dar es Salaam, with ≥2 weeks of cough or fever, or weight loss. Subjects gave 2 mLs of urine to test for LAM using a commercially available ELISA, ≥2 sputum specimens for concentrated AFB smear and solid media culture, and 40 mLs of blood for culture.

**Results:**

Among 212 evaluable subjects, 143 (68%) were female; mean age was 36 years; and the median CD4 count 86 cells/mm^3^. 69 subjects (33%) had culture confirmation of tuberculosis and 65 (31%) were LAM positive. For 69 cases of sputum or blood culture-confirmed tuberculosis, LAM sensitivity was 65% and specificity 86% compared to 36% and 98% for sputum smear. LAM test characteristics were not different in patients with bacteremia but showed higher sensitivity and lower specificity with decreasing CD4 cell count. Two month mortality was 64 (53%) of 121 with outcomes available. In multivariate analysis there was significant association of mortality with absence of anti-retroviral therapy (p = 0.004) and a trend toward association with a positive urine LAM (p = 0.16). Among culture-negative patients mortality was 9 (75%) of 12 in LAM positive patients and 27 (38%) of 71 in LAM negative patients (p = 0.02).

**Conclusions:**

Urine LAM is more sensitive than sputum smear and has utility for the rapid diagnosis of culture-confirmed tuberculosis in this high-risk population. Mortality data raise the possibility that urine LAM may also be a marker for culture-negative tuberculosis.

## Introduction

In Africa, tuberculosis causes significant morbidity and mortality, especially among people with HIV infection [1]. Early diagnosis is a high priority for tuberculosis control and to prevent mortality [2]. Since 1996, the World Health Organization has promoted the DOTS strategy for tuberculosis control, one aspect of which is case detection through sputum acid fast bacillus (AFB) smear microscopy [3]. However, the sensitivity for diagnosing pulmonary tuberculosis using direct, unconcentrated sputum smear methods ranges from 40 to 60% for a combination of three examinations [4] and is lower for those with HIV co-disease [5]. Additionally, sputum smear cannot be used for persons who cannot produce sputum such as children and those with extrapulmonary disease.

Lipoarabinomannan (LAM) is a cell wall lipopolysaccharide specific for the genus *Mycobacterium* [6], [7]. LAM is released when *M. tuberculosis* is lysed by the host immune system, filtered by the kidneys and can be detected in the urine as potential same day diagnostic test for tuberculosis. Test characteristics for the diagnosis of tuberculosis have been variable [8], and one study in patients with HIV showed an association of urinary LAM with severe tuberculosis and high mortality [9]. Theoretical advantages of urine LAM detection includes ease of specimen collection, reduced chance of nosocomial transmission during collection compared to sputum, and test performance that does not depend on an intact immune system.

The goals of the present study were to prospectively evaluate the diagnostic accuracy of urinary LAM antigen detection among HIV-infected patients hospitalized with suspect tuberculosis in Tanzania using culture positivity as the gold standard for the diagnosis of tuberculosis. In addition we assessed the prognostic value of urinary LAM for survival.

## Materials and Methods

### Patients

Eligible patients were HIV-infected adults ≥18 years old admitted to one of two participating district hospitals (A and B) in Dar es Salaam, Tanzania, and considered tuberculosis suspects based on cough or fever for at least two weeks or unexplained weight loss. Patients were prospectively identified by trained research staff attending the hospital physicians' morning report as part of a study in the same patients to determine the optimal blood culture method for the diagnosis of disseminated tuberculosis [10]. Research staff explained the purpose and the conduct of the study, and requested written informed consent in Kiswahili or in English.

### Studies

Within 72 hours of enrolment, a random 2 mL sample of urine was collected in a sterile plastic container and stored at 2–8°C within four hours for up to 24 hours before freezing or processing. At the time of enrollment, each subject was requested to provide two random and one early morning sputum samples for AFB smear microscopy and culture, and also underwent phlebotomy for repeat (confirmatory) HIV testing, CD_4_+ T-lymphocyte count, and mycobacterial blood culture. A total of 40 mLs of blood were cultured for mycobacteria by two methods : automated MB BacT broth and manual Isolator lysis-centrifugation agar. Blood for culture was collected by random assignment during one phlebotomy (40 mLs) or two phlebotomies 12–24 hours apart (20 mLs twice) to determine the optimal approach to mycobacterial blood culture [10]. Other tests were done at the discretion of the attending physician.

### Follow-up

At two months after enrollment, subjects underwent follow up at the hospital (if still inpatient), at a centrally located outpatient clinic (if discharged), or were contacted by phone if they did not return or were unable to travel to the outpatient clinic. If the subject died prior to follow up or could not be contacted, efforts were made in person or by phone to gather outcome data from next of kin, who had been identified by the subject at the time of enrollment. Urine LAM test results were not known to personnel conducting follow-up investigations. Mortality was defined as the number of confirmed deaths within two months divided by the total number of study subjects with available 2 month follow-up.

### HIV testing

Serum HIV testing was performed by trained research personnel according to the guidelines and procedures currently approved by the Tanzania Ministry of Health. Sera were evaluated for HIV by 2 serial rapid HIV tests: SD Bioline (Standard Diagnostics, Inc., Korea) and Determine (Inverness Medical, Japan). HIV testing was performed during enrolment into the study, and both tests were required to be positive for eligibility.

### Microbiology

Sputum specimens were examined for AFB using the Ziehl-Neelsen method and cultured for mycobacteria on Lowenstein Jensen slants for up to 8 weeks. Blood was cultured for mycobacteria using both an automated broth-based system (BacT/ALERT® MB) and a manual agar-based lysis centrifugation system (Wampole™ ISOSTAT®/ISOLATOR™ Microbial System) according to manufacturer's instructions (bioMérieux, Durham NC and Inverness, Waltham MA, respectively). AFB were considered *M. tuberculosis* if the AFB positive isolate had typical colonial morphology

### Urine LAM test

The urine LAM test ELISA used in this study was initially marketed as the MTB-LAM ELISA (Chemogen Inc., South Portland ME). Mid-study, the test name was changed and marketed as Clearview TB ELISA (Inverness Medical Innovations, Waltham MA) but methods were unchanged. Urine LAM testing was conducted according to manufacturer instructions by a single research laboratorian who was unaware of the results of other diagnostic tests. A 0.5–2 ml aliquot of urine was heated at 95–100°C for 30 minutes, cooled to room temperature then centrifuged at 10,000 rpm for 15 min. Duplicate supernatant samples per subject were refrigerated and tested by ELISA on batches (n = 10), or were frozen at −20°C and tested by ELISA in batches (all other samples). Duplicate sample results were averaged, and the summary interpretation was considered positive when the optical density (OD) at 450 nm was at least 0.1 above the average signal of the negative control. Urine LAM test results were not provided to treating clinicians.

### Analysis

A subject with tuberculosis was defined by at least one sputum or blood culture positive for *M. tuberculosis*. A subject without tuberculosis was defined by having provided at least two sputum specimens with associated negative cultures, and negative blood culture (if blood was obtained for culture). Sensitivity, specificity, and positive and negative predictive values of the urine LAM test were calculated if there was a valid urine LAM test using SPSS and Excel.

Statistical significance was determined in univariate analyses using Chi-square for categorical variables and the Mann-Whitney test for non-normally distributed variables. Risk ratios and 95% confidence intervals were estimated using generalized linear models assuming a binomial distribution with log-link function. An ROC curve for different OD cutoffs of the LAM urine test was generated using a web-based calculator [11].

### Human subjects approval

Human subjects research ethical review for the study was obtained from an Institutional Review Board of the Dartmouth Medical School (Hanover NH USA), the National Institute for Medical Research (Dar es Salaam, Tanzania), and the Muhimbili University of Health and Allied Sciences Ethics Review Board. Written informed consent was obtained from all subjects prior to enrolment into the study. The respective institutional review boards approved the informed consent form. Subjects did not receive monetary incentive for participation

## Results

### Tuberculosis

Between May 2007 and July 2008, 278 TB suspects were approached and requested to participate in the study, 271 patients (97%) agreed to participate. Of the 7 who did not participate the most common reason was concern for the volume of blood that would be drawn. Of the 271 who agreed to participate 13 patients were HIV negative and were excluded from the study. An additional 46 subjects were excluded because they were too weak to produce urine for LAM testing. Of the remaining 212 subjects, the median age was 36 years (range 18–65); 143 (67%) were female, median CD4 count 86 (range 1–1016) and 68 patients (32%) were on anti-retroviral therapy (ART) at enrollment (mean duration of ART = 324 days). One hundred twelve were from hospital A and 100 were from hospital B; there was no significant difference in age or sex distribution between the hospital cohorts. Tuberculosis was diagnosed by culture in 69 of 212 study subjects (33%).

### Urine LAM

Urine LAM was positive in 65 (31%) subjects including 45/69 (65%) of patients with culture-confirmed tuberculosis and 20/143 (14%) of patients without culture-confirmed tuberculosis. The sensitivity and specificity of a positive LAM for culture-confirmed tuberculosis were 65% and 86% respectively (Table 1) compared to the sensitivity and specificity of sputum smear for culture confirmed TB that was 36% and 98% for sputum smear respectively. LAM sensitivity was higher and specificity lower with decreasing CD4 cell count. Sensitivity was not increased in patients with disseminated tuberculosis as defined by a positive blood culture. In the sample of eight specimens on which dilutions were performed, we observed consistent results in 88% (7/8, data not shown). LAM positive rates were 2 (20%) of 10 fresh samples versus 63 (31%) of 202 frozen samples (p = 0.69).

**Table 1:** Urine LAM test characteristics for 69 cases of culture-confirmed tuberculosis among 212 tuberculosis suspects.

	Sensitivity (%)	Specificity (%)	PPV (%)	NPV (%)
**Culture-confirmed tuberculosis, site**				
**Sputum culture positive (all)**	41/57 (72%)	116/132 (88%)	41/57 (72%)	116/132 (88%)
**[95% CI]**	[58%–83%]	[81%–93%]	[58%–83%]	[81%–93%]
**Sputum culture positive (positive smear)**	17/23 (74%)	105/118 (89%)	17/30 (57%)	105/111 (95%)
**[95% CI]**	[53%–89%]	[82%–94%]	[39%–73%]	[89%–98%]
**Blood culture positive**	20/31 (65%)	136/181 (75%)	20/65 (31%)	136/147 (93%)
**[95% CI]**	[45%–80%]	[68%–81%]	[20%–44%]	[87%–96%]
**Any culture positive**	45/69 (65%)	123/143 (86%)	45/65 (69%)	123/147 (84%)
**[95% CI]**	[53%–76%]	[79%–91%]	[56%–80%]	[76%–89%]
**Culture-confirmed tuberculosis, CD4 strata**				
**CD4<50**	24/31 (77%)	31/43 (72%)	24/36 (67%)	31/38 (82%)
**[95% CI]**	[58%–89%]	[56%–84%]	[49%–81%]	[65%–92%]
**CD4 50–200**	14/21 (67%)	32/34 (94%)	14/16 (88%)	32/39 (82%)
**[95% CI]**	[43%–85%]	[79%–99%]	[60%–98%]	[66%–92%]
**CD4>200**	4/13 (31%)	43/49 (88%)	4/10 (40%)	43/62 (69%)
**[95% CI]**	[10%–61%]	[75%–95%]	[14%–73%]	[56%–80%]

PPV = positive predictive value; NPV = negative predictive value.

**Figure 1:**
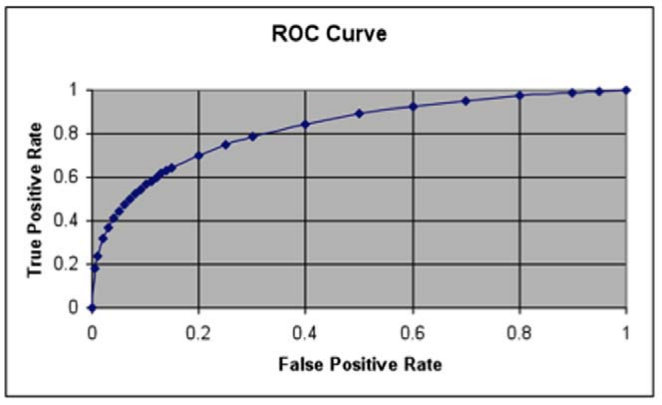
ROC curve assessing optical density of LAM result as a predictor of culture-confirmed tuberculosis in a cohort of 212 HIV-infected, hospitalized patients in Dar es Salaam, Tanzania. The area under the curve is 0.828.

A Receiver Operating Characteristic (ROC) curve to illustrate how different OD cutoffs affect sensitivity and specificity of the urine LAM is presented in Figure 1. The area under the ROC curve was 0.83, indicating moderate test accuracy.

### Mortality

**Figure 2:**
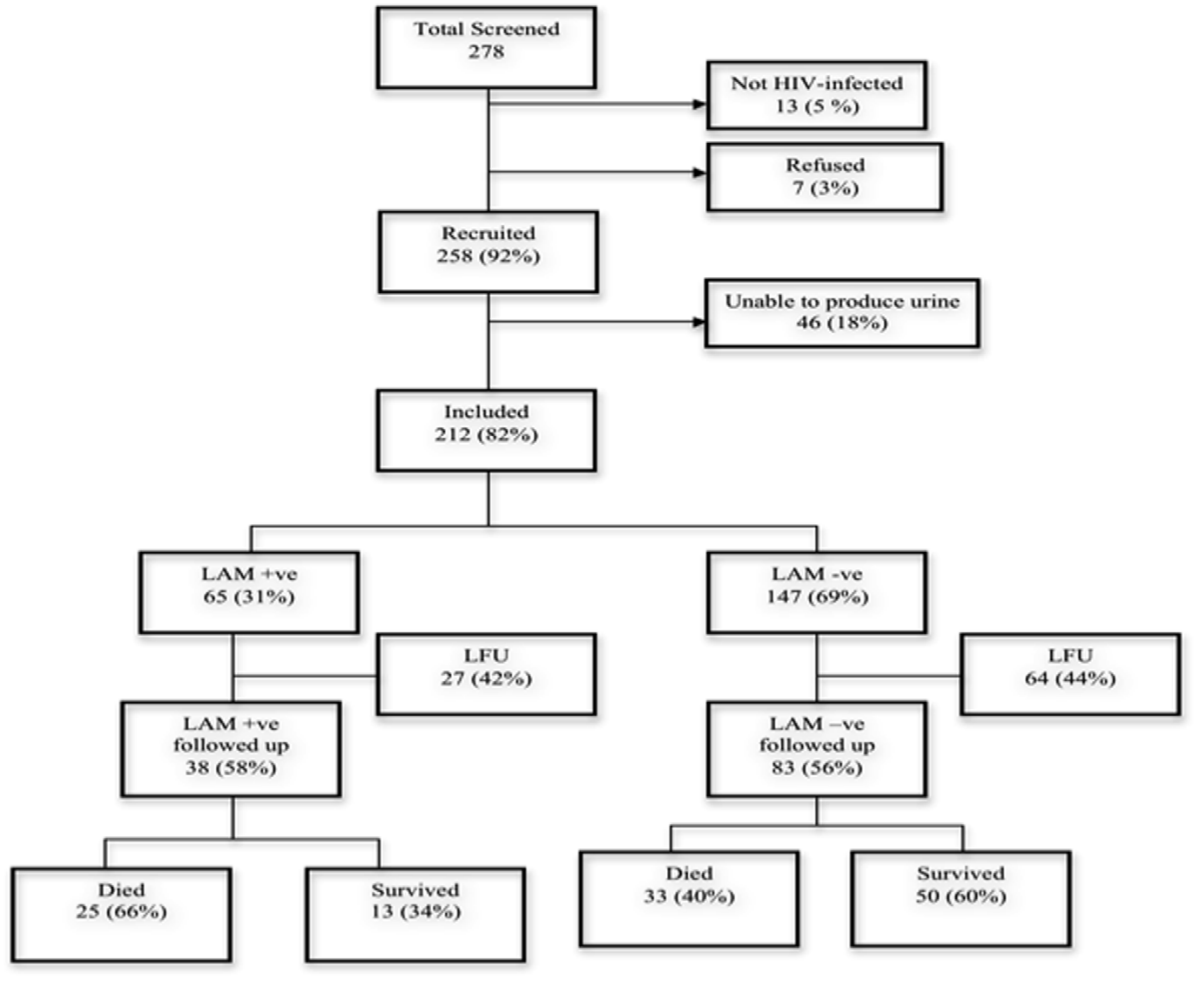
Mortality by LAM result.

A total of 121 (57%) of 212 subjects had follow up information available at 2 months, including 38 (55%) of the subjects with confirmed tuberculosis and 83 (58%) of the subjects without confirmed tuberculosis (p = 0.68). The remaining subjects did not return for follow-up and could not be reached by phone. Sixty four (53%) of 121 subjects with follow-up data died; 38 of these deaths occurred during the enrollment hospital admission. Univariate risk factors for mortality are shown in Table 2 and risk ratios in Table 3. Significant associations with mortality (p<0.05) included CD4 count, absence of anti-retroviral therapy (ART) and positive urine LAM test. As shown in Figure 2, among those with follow up available, mortality was 25 (66%) of 38 subjects with a positive urine LAM test and 33 (40%) of 83 subjects with a negative urine LAM test (p = 0.011). In multivariate analysis, there was a significant association between mortality and absence of ART and a trend towards association with a positive LAM (Table 4). Among culture-negative patients mortality was 9 (75%) of 12 in LAM positive patients and 27 (38%) of 71 in LAM negative patients (0.016). TB treatment data was available on 6 of 20 LAM-positive, culture-negative patients: among 3 survivors all 3 had received treatment for TB; among 3 of 9 fatal cases with data, 2 had received treatment for TB.

**Table 2:** Risk factors for mortality among 121 tuberculosis suspects with follow-up data.

Characteristic	Died	Survived	P value
	n = 58	n = 63	
**Age, median**	37 (22–65)	38 (18–56)	0.691
**Sex, Female %**	39 (67%)	42 (67%)	1.000
**CD4, median**	30 (2–853)	106 (1–849)	0.014
**On ART** * **, %**	8 (14%)	29 (46%)	<0.001
**Any TB culture positive**	22 (38%)	16 (25%)	0.099
**Blood culture positive**	15 (26%)	1 (2%)	<0.001
**Urine LAM positive**	25 (43%)	13 (21%)	0.007

* ART means the patient was on ART at enrollment.

**Table 3:** Univariate analysis of mortality and risk factors among 121 tuberculosis suspects with follow-up data.

Characteristic	Died (%)	Risk Ratio	P value
**Female**	39 (48.1%)		
**Male**	19 (47.5%)	1.01	1.000
**CD4>200**	8 (29.6%)		
**CD4≤200**	43 (55.1%)	1.86	0.027
**On ART** *	8 (21.6%)		
**Not on ART**	50 (59.5%)	2.75	<0.001
**TB culture negative**	36 (43.4%)		
**TB culture positive**	2 (57.9%)	1.33	0.171
**Blood culture negative**	43 (41.0%)		
**Blood culture positive**	15 (93.8%)	2.29	<0.001
**Negative LAM**	33 (39.8%)		
**Positive LAM**	25 (65.8%)	1.65	0.011

* On ART means at the time of enrollment.

**Table 4:** Multivariate analysis of mortality and risk factors among 121 subjects with suspect tuberculosis and follow-up data.

Characteristic	Risk ratio	95% CI	P value
**CD4>200**	1		
**CD4≤200**	1.499	0.830–2.709	0.180
**On ART**	1		
**Not on ART**	2.998	1.422–6.320	0.004
**Negative LAM**	1		
**Positive LAM**	1.271	0.907–1.781	0.163

## Discussion

In a population of hospitalized subjects with advanced HIV infection and suspect tuberculosis we found that urine LAM testing was almost twice as sensitive as AFB smear for the rapid diagnosis of tuberculosis. Superior sensitivity is likely due in part to the high rate of disseminated and extra-pulmonary disease where sputum smear and/or culture may be negative but mycobacterial antigen may still be circulating, filtered and detectable in urine. Specificity of LAM was lower than for AFB smear, but this can be considered an acceptable limitation in this patient population with a high short term mortality from tuberculosis that is often undiagnosed or untreated [10], [12].

The LAM test characteristics we identified in the present study are consistent with those described in a systematic review of seven studies that assessed test accuracy using only microbiologically confirmed cases (such as ours): sensitivity was 13%–93% and specificity 87–99% [8]. Differences in test characteristics may relate both to different LAM testing methodologies and different patient populations. In the systematic review pooled sensitivity estimates from the two studies that evaluated the early prototype version of the test were significantly higher than estimates from studies that used either of the two commercial assays [8]. Urine collection and processing may influence accuracy, although analysis of sub-groups in which the urine used in the assay was either fresh or previously frozen found no statistically significant differences between these groups [8]. In our study, most assays were conducted on frozen samples and we had too few results on fresh samples for comparison.

The effect of different patient populations is most evident with HIV where sensitivity has been found to be higher in HIV-positive than in HIV-negative patients and higher with decreasing CD4 count, as we also observed [8], [13]. This could reflect a higher circulating burden of *M. tuberculosis* in advanced AIDS as suggested by a study that showed higher sensitivity with positive blood cultures [9]. We did not find higher sensitivity with positive blood cultures. This could reflect different levels of mycobactermia detected by different blood culture methods, and could be resolved with a blood culture study comparing colony forming units (CFUs) in positive blood cultures with LAM results.

Subjects with a positive urine LAM had significantly greater two-month mortality in univariate analysis and a trend toward greater mortality in multivariate analysis. Further, mortality among culture-negative subjects was significantly higher in LAM positive than LAM negative subjects. This is concordant with the findings of a South African study of hospitalized patients in whom positive LAM results were associated with greater mortality among culture-confirmed tuberculosis cases [9]. These findings raise the intriguing possibility that urine LAM may not only be a marker for more severe culture-positive disease but may also be a marker for culture-negative tuberculosis, incipient tuberculosis, extra-pulmonary tuberculosis, or perhaps another unindentified opportunistic infection. Since we did not have follow-up on all subjects, and since rigorous statistical significance was not achieved in the multivariate analysis with our small sample size, these findings require confirmation in a larger study.

Our data suggest a role for LAM testing in hospitalized HIV-infected patients with suspect tuberculosis who have a very high short-term risk of mortality. Urine LAM detected 24 patients who were smear negative and culture-confirmed and 4 patients who could not produce sputum for evaluation. This supports the use of urine LAM testing combined with AFB sputum smear microscopy for early initiation of treatment in patients with CD4 counts <200 as suggested by Peter et al [14]. In addition HIV-positive patients with this profile should also be started on anti-retroviral therapy within 8 weeks, and those with a positive LAM or CD4<50 within 2 weeks [15].

We have avoided many biases seen in studies of diagnostics [16]. The spectrum of patients is representative of the patients who will receive the test in practice (no spectrum composition bias), the reference standard is independent of the index test (incorporation bias), and tests were interpreted without knowledge of other test results (reference standard bias). We have also attempted to meet all STARD (STAndards for the Reporting of Diagnostic accuracy studies) recommendations in this report [17]. Yet, our study has limitations. A significant number of screened subjects were unable to produce urine. Culture for *M. tuberculosis* was performed on agar, which is less sensitive than broth, a factor which may have lowered the apparent sensitivity and specificity of LAM testing, and may also explain some of the culture-negative LAM positive cases (non-tuberculous mycobacteria would have been isolated with our culture methods). Further, although there was no evident systematic bias in our attempts to obtain two-month follow-up data, we cannot exclude this possibility since we only had these data in slightly more than half of the study population. Finally the small sample size makes it difficult to fully separate the influence of related variables on mortality such as positive LAM and positive culture, or CD4 and ART.

Urine LAM testing is a promising rapid diagnostic for suspect tuberculosis among hospitalized patients with HIV infection living in a tuberculosis-endemic country. The suggestive association of a positive urine LAM with mortality raises the possibility that urine LAM may have a role in the diagnosis of culture-negative tuberculosis.
